# Bone mineral density saturation as influenced by the visceral adiposity index in adults older than 20 years: a population-based study

**DOI:** 10.1186/s12944-023-01931-y

**Published:** 2023-10-06

**Authors:** Zi-hao Chen, Ting-feng Zhou, Yi-tian Bu, Lei Yang

**Affiliations:** 1 Department of Orthopaedic Surgery, The Second Affiliated Hospital, Yuying Children Hospital of Wenzhou Medical University, Wenzhou, 325000 China; 2 Department of Otolaryngology, First Affiliated Hospital of Wenzhou Medical University, Wenzhou, 325000 China

**Keywords:** Visceral adiposity index, Bone mineral density, Osteoporosis, Obesity, National Health and Nutrition Examination Survey

## Abstract

**Objective:**

The goal of this research was to determine whether or not there is a saturation effect and whether or not the visceral adiposity index (VAI) correlates with bone mineral density (BMD) in adult Americans.

**Methods:**

This study used multivariate logistic regression models to examine the association between VAI and total femur BMD, drawing on the most up-to-date data from the National Health and Nutrition Examination Survey (NHANES) between 2007 and 2018. Saturation levels and non-linear connections were calculated using a smooth curve-fitting algorithm and an investigation of saturation effects. Subgroup analyses and interaction tests were also conducted.

**Results:**

This study ultimately recruited 6257 individuals aged 20 years or older. According to multivariate regression analysis, those with high VAI scores exhibited higher total femur BMD. Total femur BMD was greater in the highest VAI quartile (Q4: 0.060 g/cm^2^) after adjustment than in the lowest VAI quartile (Q1) (*P* < 0.05). After controlling for variables, subgroup analysis failed to reveal any significant interaction effects. Furthermore, the study determined that VAI and BMD exhibited a specific saturation effect through the investigation of the saturation effect and the fitting of smooth curves. Saturation effect investigation of total femur BMD using VAI revealed a saturation value of 3.3.

**Conclusion:**

The present study uncovered a non-linear relationship between VAI and total femur BMD, which exhibited a saturation effect.

## Background

Reduced bone mass and microstructural degradation in bone tissue define osteoporosis, a systemic skeletal condition that increases the risk of fracture [1]. Previous studies have demonstrated an increased prevalence of osteoporosis among middle-aged and elderly individuals each year [2, 3]. As the world’s population ages, osteoporosis significantly impacts the economy and public health [4]. Bone mineral density (BMD) is a reliable indicator of osteoporosis, and low BMD is related to a higher risk of fracture [5]. Thus, the search for novel risk factors for low BMD in osteoporosis is gaining more attention and is expected to lead to new preventative approaches.

By combining high-density lipoprotein (HDL), triglycerides (TG), body mass index (BMI), and waist circumference (WC), one may reliably predict visceral fat accumulation and adipose tissue dysfunction using the Visceral Adiposity Index (VAI) [6, 7]. It is a novel and one-of-a-kind biomarker for measuring visceral adipose function indirectly [8]. VAI is superior to conventional measures of adiposity, like BMI and WC, in differentiating between subcutaneous and visceral fat [9]. Visceral obesity may be detected with great accuracy using computed tomography (CT) and magnetic resonance imaging (MRI).

Obesity is a major global health issue affecting individuals worldwide [10]. Previous studies have shown that obesity enhances BMD due to increased mechanical stress, which may aid bone preservation [11, 12]. However, extreme obesity has significant negative impacts on various organs and systems, including type 2 diabetes [13], atherosclerosis [14], and non-alcoholic fatty liver disease. The current research tested the hypothesis that VAI reaches a saturation threshold and that sustaining VAI at this level results in a healthy compromise between obesity and BMD. Therefore, it is essential for public health to establish the VAI that would strike a happy medium between obesity and BMD.

Expanding on the theoretical framework outlined earlier, this study delved into the impact of VAI on total femur BMD, and explored the existence of a saturation point between the two variables. Drawing on extensive demographic data sourced from the National Health and Nutrition Examination study (NHANES) database, this research offers fresh perspectives on the underlying mechanisms at play.

## Methods

### Research subjects

**Fig. 1:**
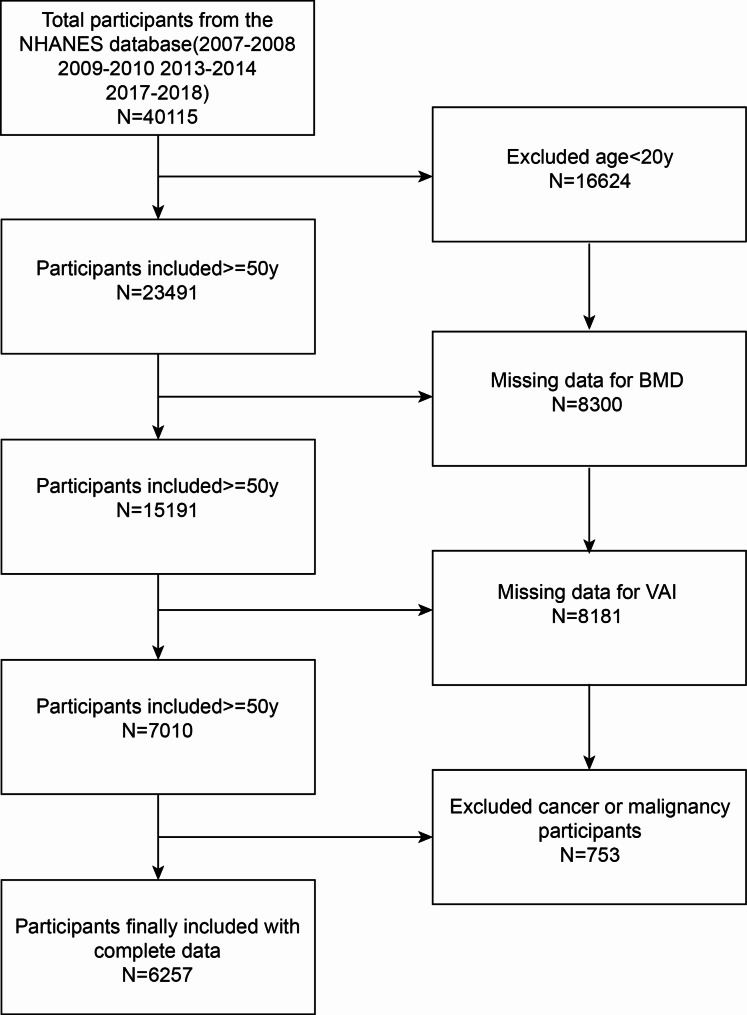
Flowchart illustrating participant selection. Legend: VAI, visceral adiposity index; BMD, bone mineral density; NHANES, National Health and Nutrition Examination Survey

The NHANES is a large, continuing cross-sectional study in the United States with the goal of collecting accurate data on health-related topics and addressing new public health challenges. To examine the relationship between nutrition and health in the United States, this research relied only on data collected from NHANES, laboratory components, and interviews. The present study collected data from NHANES 2007–2018, excluding NHANES 2011–2012 and NHANES 2015–2016, since BMD data were unavailable during those periods. The present study meticulously applied inclusion and exclusion criteria to arrive at a refined sample size of 6257 participants. Specifically, 16,624 participants under the age of 20, 8300 participants without BMD data, 8181 participants without VAI data, and 753 individuals with malignancy or cancer were excluded. This rigorous approach ensures the robustness and reliability of the study findings (Fig. 1).

### Outcome and exposure factors

The major outcome indicator of this study was the evaluation of total femur BMD using dual-energy X-ray absorptiometry (DXA). VAI was the primary risk factor, and it was calculated in the following ways depending on the person’s gender: VAI = WC/[39.68 + (BMI1.88)] * (TG/1.03) * (1.31/HDL) for men, and VAI = WC/[36.58 + (BMI1.89)] * (TG/0.81) * (1.52/HDL) for women. Calculations were made in mmol/L for TG and HDL, cm for WC, and kg/m^2^ for BMI.

### Covariates

The following covariates were modified to strengthen the relationship between total femur BMD and VAI: smoking status, education level, race, gender, age, moderate activities, diabetes, creatinine, blood urea nitrogen, alkaline phosphatase (ALP), alanine transaminase (ALT), the ratio of family income to poverty, aspartate aminotransferase (AST), phosphorus, total cholesterol, total calcium, and total protein. This study also used pre-specified effect modifiers to assess the interaction impact and considered age (< 60/≥60 years), gender (male/female), and diabetes (yes/no) as stratified variables.

### Statistical analysis

In NHANES, sampling weights are frequently utilized to consider more intricate research designs. Continuous variables were represented by means and standard deviations (SDs), whereas proportions were used to display categorical data. The data was analyzed using a chi-square test and a weighted t-test for significance. Using logistic regression models, we looked at the association between VAI and total femur BMD, both with and without controlling for potential confounding factors. Model 1 was not tweaked in any way. The second model took demographic factors into account, including age, gender, and race. Multiple factors i.e., age, gender, race, education, smoking, moderate activity, diabetes, family income-to-poverty ratio, alanine aminotransferase, aspartate aminotransferase, blood urea nitrogen, creatinine, phosphorus, total calcium, total protein, and total cholesterol were all included into Model 3. Multiple sensitivity analyses and propensity score matching were used to further examine the connection between VAI and total femur BMD. This research used the generalized additive model (GAM) and curve fitting to further examine whether or not VAI was associated with the risk of low total femur BMD. The upward trend in BMD was found to slow as VAI rises, with BMD eventually leveling off once VAI reaches a specific threshold, known as the saturation effect. Values for inflection points were established by likelihood ratio tests after it was shown that a non-linear relationship existed. Finally, subgroup analyses were stratified by sex, age, race, and diabetes status using hierarchical logistic regression models. Although power analysis was not employed in this work, based on our findings from other research, we considered the current sample size to be adequate [15–17]. All statistical analyses were conducted using R and Empower Stats. A two-tailed *P*-value of less than 0.05 was considered statistically significant.

## Results

### Baseline features

The research included a total of 6257 individuals that were eligible to participate. 49.46% of participants were women, while 50.54% were men overall. The VAI was considered both a continuous independent variable and a categorical variable (split into quartiles), with the lowest quartile acting as the benchmark. Among different groups of VAI (quartiles, Q1–Q4), age, race, education level, smoking status, moderate activities, diagnosed diabetes, family poverty ratio/median income, ALT, ALP, AST, phosphorus, blood urea nitrogen, total protein and total cholesterol, and total femur BMD are all significantly different (Table 1).

**Table 1:** Sample characteristics weighted for the research

Visceral adiposity index	Quartile1 (<0.90)	Quartile2 (0.90–1.45)	Quartile3 (1.45–2.43)	Quartile4 (>2.43)	*P*-value
	N = 1562	N = 1559	N = 1568	N = 1568	
**Age (year)**	48.437 ± 15.714	49.878 ± 15.585	50.804 ± 15.744	51.487 ± 14.574	< 0.00001
**Gender(%)**					0.70799
Male	49.964	49.854	48.814	50.943	
Female	50.036	50.146	51.186	49.057	
**Race(%)**					< 0.00001
Mexican American	5.686	7.337	10.567	10.788	
Other Hispanic	4.440	5.915	6.188	6.550	
Non-Hispanic White	64.674	69.232	66.231	69.574	
Non-Hispanic Black	17.490	10.751	8.874	4.927	
Other Race - Including Multi-Racial	7.710	6.766	8.141	8.161	
**Education level(%)**					< 0.00001
Less than 9th grade	4.398	5.031	6.578	8.277	
9-11th grade	9.358	10.306	12.950	15.147	
High school graduate	21.213	23.704	24.154	27.559	
Some college or AA degree	27.860	29.408	30.550	29.419	
College graduate or above	37.155	31.378	25.695	19.473	
**Smoked at least 100 cigarettes(%)**					< 0.00001
Yes	39.581	44.019	46.672	52.045	
No	60.389	55.981	53.328	47.845	
**Moderate activities(%)**					< 0.00001
Yes	53.219	45.279	42.290	36.774	
No	46.781	54.721	57.710	63.226	
**Diagnosed diabetes** **(%)**					< 0.00001
Yes	5.456	6.199	11.068	16.063	
No	93.207	91.961	86.199	80.229	
Ratio of family income to poverty	3.180 ± 1.634	3.174 ± 1.639	2.974 ± 1.618	2.841 ± 1.627	0.00001
ALT (U/L)	23.578 ± 22.773	23.635 ± 13.679	25.392 ± 15.283	29.135 ± 19.956	< 0.00001
ALP (U/L)	63.843 ± 21.543	66.892 ± 21.588	70.168 ± 21.739	72.932 ± 25.882	< 0.00001
AST (U/L)	26.349 ± 23.851	24.493 ± 17.757	24.469 ± 11.560	26.489 ± 17.582	0.00057
Blood urea nitrogen (mmol/L)	4.830 ± 1.751	4.774 ± 1.751	4.726 ± 1.869	4.985 ± 2.078	0.00080
Creatinine (µmol/L)	77.542 ± 28.015	77.479 ± 21.326	77.980 ± 32.071	78.150 ± 29.516	0.88565
Phosphorus (mmol/L)	1.195 ± 0.174	1.172 ± 0.172	1.155 ± 0.178	1.181 ± 0.175	< 0.00001
Total calcium (mmol/L)	2.339 ± 0.082	2.344 ± 0.088	2.342 ± 0.089	2.346 ± 0.087	0.13385
Total protein (g/dL)	7.085 ± 0.463	7.069 ± 0.437	7.101 ± 0.439	7.144 ± 0.443	0.00002
Total Cholesterol (mmol/L)	4.806 ± 0.926	4.950 ± 0.962	5.069 ± 1.069	5.379 ± 1.201	< 0.00001
Triglyceride (mmol/L)	0.668 ± 0.193	1.030 ± 0.219	1.418 ± 0.307	2.700 ± 2.159	< 0.00001
HDL-C (mmol/L)	1.787 ± 0.422	1.482 ± 0.319	1.280 ± 0.271	1.064 ± 0.239	< 0.00001
LDL-C (mmol/L)	2.713 ± 0.772	2.995 ± 0.843	3.139 ± 0.962	3.147 ± 1.000	< 0.00001
BMI (kg/m^2^)	25.526 ± 5.156	27.530 ± 5.381	29.494 ± 5.685	30.614 ± 5.538	< 0.00001
Waist Circumference (cm)	90.035 ± 13.466	95.962 ± 13.599	101.192 ± 13.894	104.786 ± 13.047	< 0.00001
Total femur bone mineral density (g/cm^2^)	0.949 ± 0.157	0.953 ± 0.159	0.970 ± 0.155	0.988 ± 0.156	< 0.00001

Note: For continuously varying variables, mean +/- SD: By using a weighted linear regression model, the *P*-value was computed

% is a category variable: Using the weighted chi-square test, the *P*-value was determined

### Association between visceral adiposity index and total femur BMD

**Fig. 2:**
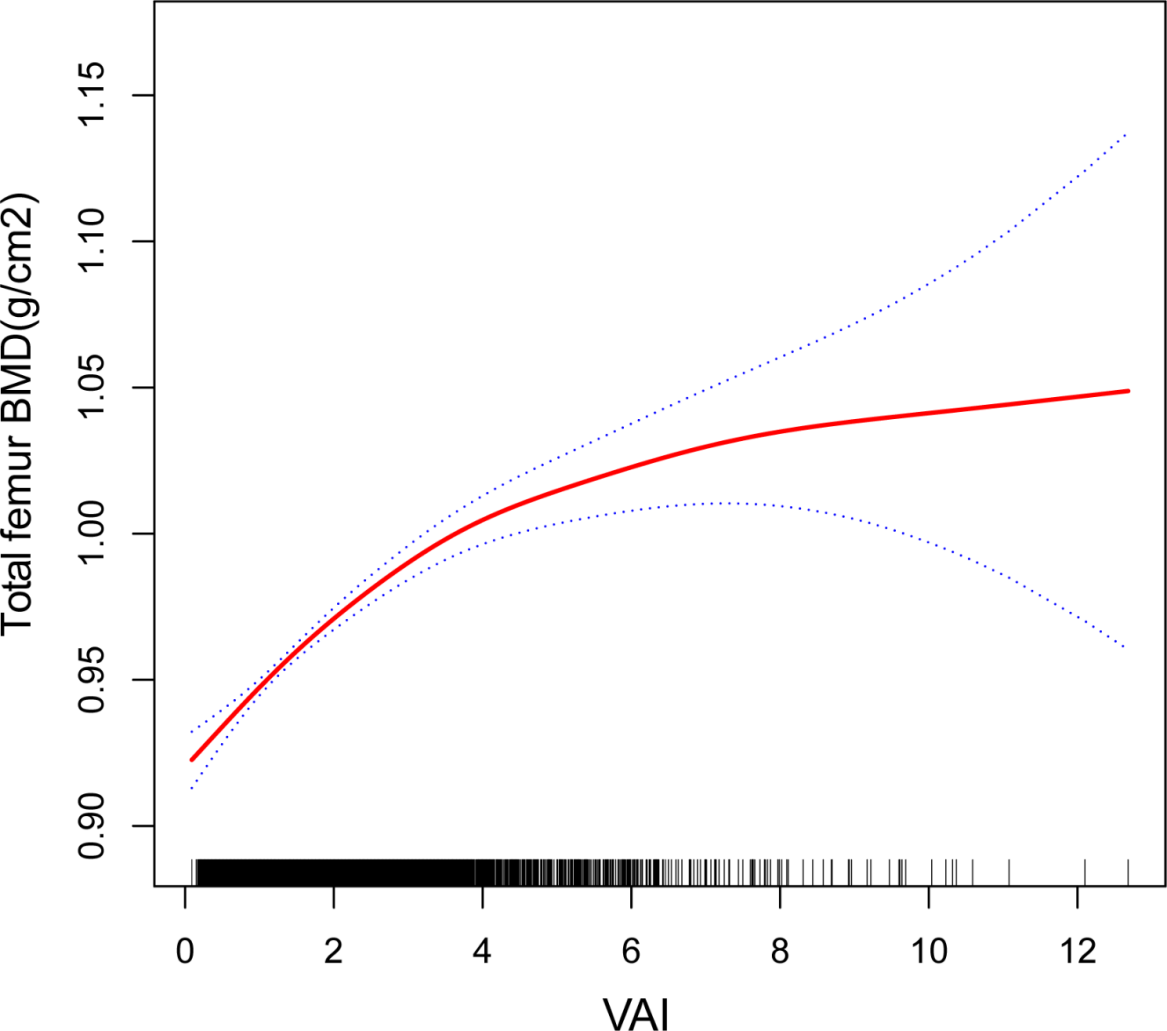
The relationship between the visceral adiposity index and the total bone mineral density of the femur. Legend: The smooth red line indicates the best possible fit of the curve between the variables. The blue shading indicates the 95% CI for the fit. All potential confounds were eliminated

Model 1 [β(95%CI) = 0.002 (0.001, 0.003)], Model 2 [β(95%CI) = 0.003 (0.002, 0.004)], and Model 3 [β(95%CI) = 0.016 (0.014, 0.019)] of multivariate regression analysis revealed a positive correlation between VAI and total femur BMD. When VAI was transformed from a continuous to a categorical variable (quartiles), Model 1 [0.026(0.015,0.037)], Model 2 [0.054(0.044,0.064) < 0.00001], and Model 3 [0.060(0.049,0.071) < 0.00001] all found that Q4 participants had significantly higher total femur BMD than Q1 participants. There were no significant trends (*P* < 0.05) across any of the three models (Table 2). When employing smooth curve fitting, GAM, and piecewise linear regression, a connection between VAI and total femur BMD was investigated (Fig. 2**and** Table 3). After total adjustment, the smooth curve revealed a non-linear relationship between VAI and total femur BMD (Fig. 2). The total femur bone mineral density (BMD) exhibited a parabolic increase with an increase in visceral adiposity index (VAI), but eventually plateaued as VAI reached a certain threshold. To identify this turning point value of VAI, the present study utilized piecewise linear regression (Table 3). Total femur BMD increased by 0.025 g/cm^2^ for every unit increased in VAI when VAI reached < 3.3. The results of the study revealed a positive and non-linear correlation between VAI and total femur BMD. The saturation value was determined to be 3.3, as evidenced by a Log likelihood ratio test with a significance level of less than 0.05.

**Table 2:** Association of VAI with total femur BMD.

	Crude model (Model 1) β(95%CI) *P* value	Minimally adjusted model (Model2) β(95%CI)*P* value	Fully adjusted model (Model3) β(95%CI)*P* value
VAI	0.002 (0.001, 0.003)0.00007	0.003(0.002,0.004) < 0.00001	0.016(0.014,0.019) < 0.00001
VAI (Quartile)			
Q1	Reference	Reference	Reference
Q2	0.001(-0.011, 0.012)0.91246	0.017(0.007, 0.026) 0.00081	0.014(0.004, 0.024) 0.00674
Q3	0.011(-0.000,0.022) 0.05474	0.037(0.027,0.046) < 0.00001	0.034(0.023,0.044) < 0.00001
Q4	0.026(0.015,0.037) < 0.00001	0.054(0.044,0.064) < 0.00001	0.060(0.049,0.071) < 0.00001
*P* for trend	< 0.00001	< 0.00001	< 0 0.00001

Note: Model 1: No adjustment for covariates

Model 2: Race, gender, and age were adjusted

Model 3: Race, gender, age, education level, smoked at least 100 cigarettes, moderate activities, the doctor told you to have diabetes, family poverty ratio/median income, blood urea nitrogen, Aspartate aminotransferase, Alkaline phosphatase, Alanine aminotransferase, creatinine, phosphorus, total cholesterol, total calcium, and total protein were adjusted

**Table 3:** Analysis of the VAI saturation effect and the total BMD of the femur (g/cm^2^)

Model	Total femur BMD Adjustedβ(95%CI) *P* value
**Model1**	
the standard linear mode	0.018 (0.015, 0.020) < 0.0001
**Model2**	
Turning point (K)	3.3
VAI < 3.3	0.025 (0.021, 0.030) < 0.0001
VAI > 3.3	0.006 (0.000, 0.012) 0.0401
Log likelihood ratio test	< 0.001

Note: All models were adjusted for race, gender, age, education level, smoked at least 100 cigarettes, moderate activities, the doctor told you to have diabetes, family poverty ratio/median income, blood urea nitrogen, Aspartate aminotransferase, Alkaline phosphatase, Alanine aminotransferase, creatinine, phosphorus, total cholesterol, total calcium, and total protein

### Subgroup analysis

**Fig. 3:**
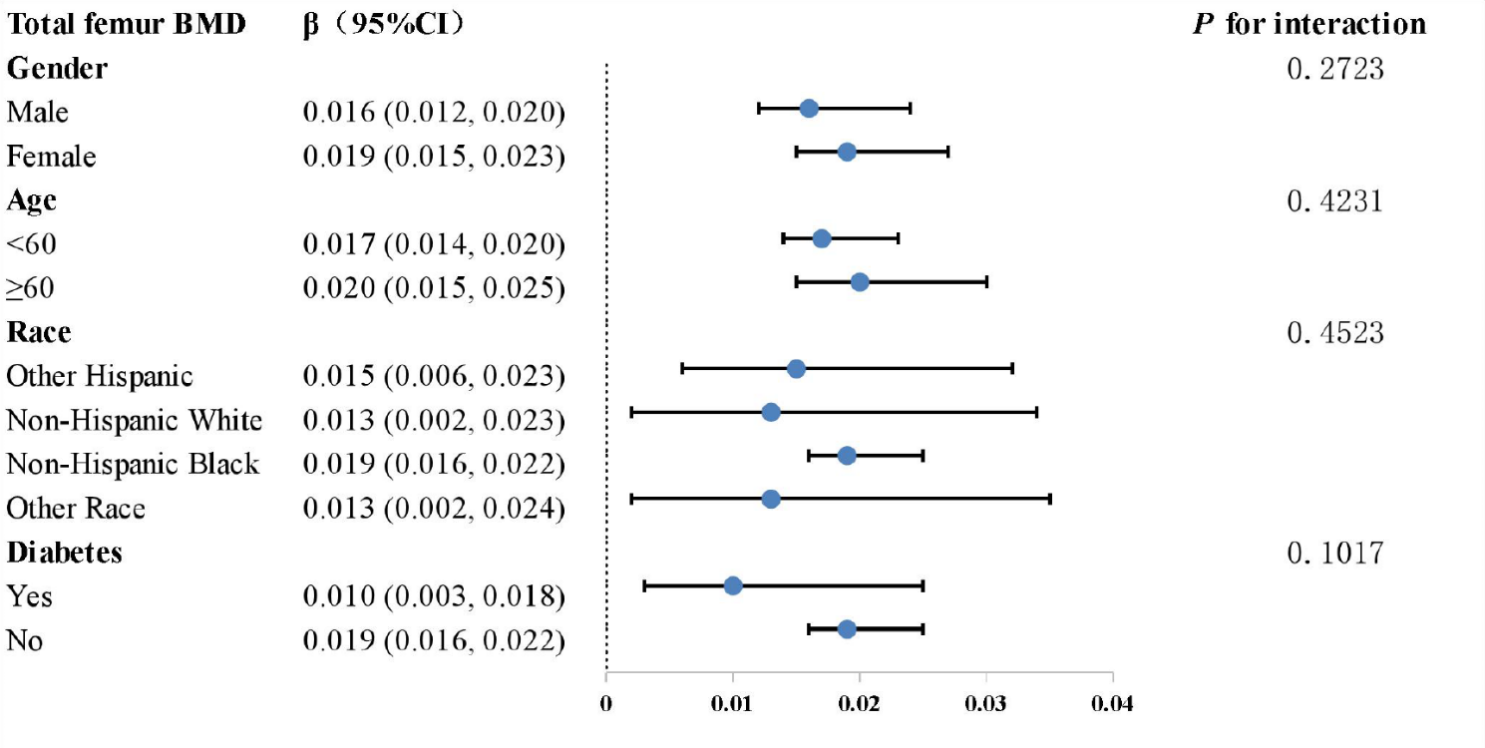
Analysis of VAI subgroups in relation to total femur BMD

The connection between VAI and total femur BMD was investigated utilizing subgroup analysis to determine whether it was stable across different demographic settings. Total femur BMD was not shown to be dependent on VAI in this study. Figure 3 illustrates that total femur BMD has a favorable relationship with VAI and was unaffected by any stratifications, including gender, age, race, and diabetes status (P values for interactions below 0.05 were consistent across the board). Among several subgroups, strong evidence of a favorable association were observed. For example, in diabetes, the present study found that each unit increases in VAI was associated with higher total femur BMD levels by 0.010 g/cm^2^. This association persisted in the absence of diabetes (β = 0.019, 95% CI: 0.016–0.022)

## Discussion

This cross-sectional analysis of 6,257 participants showed a positive relationship between VAI and total femur BMD. Notably, a VAI saturation value (3.3) in the total femur BMD for all subjects was found. As VAI increased beyond this point, the degree of the boost in total femur BMD naturally slowed, which is critical for maintaining BMD at an ideal level. Therefore, the present study considers VAI a practical indicator for clinically evaluating total femur BMD.

Osteoporosis is a biochemical condition that causes bones to weaken and become more brittle over time, increasing the likelihood that they may break [18]. A low BMD is a crucial diagnostic sign of osteoporosis. The World Health Organization (WHO) specifies a BMD of 2.5 SDs or less below the mean maximum BMD as indicative of osteoporosis [19]. The present study found a positive correlation between total femur BMD and visceral adiposity measured by VAI. Obesity is characterized by alterations in adipose tissue distribution and increased body mass, with two main types: visceral obesity, which refers to excess fat accumulation in the abdominal area, and subcutaneous obesity, which refers to fat accumulation beneath the skin [20]. However, several studies have shown that after controlling for other confounders, visceral adiposity evaluated by CT has a stronger association with BMD than subcutaneous adiposity [21–23]. VAI is a highly reliable and accurate tool for measuring the level of visceral fat in the body, making it a superior predictor when compared to other methods [8, 9, 24]. Visceral fat is closely linked to overall health in most individuals, and by providing a more precise measurement, VAI can offer valuable insights into an individual’s health status.

Both obesity and osteoporosis have become epidemics worldwide, although it is not yet clear if the two are linked. Previous research indicated that the lumbar spine and femoral neck bone densities of obese people were higher than those of normal-weight persons [12]. However, there is a variety of indicators used to evaluate obesity, particularly when it comes to the acknowledged harm of intra-abdominal fat. BMI, one of the most widely used anthropometric measures to assess obesity, was found to be positively correlated with BMD in several studies [25, 26]. However, for WC, an indicator for assessing abdominal obesity, according to certain research, WC is correlated negatively with BMD [17], supported by related studies [16, 27, 28]. Additionally, visceral adipose tissue has been demonstrated to negatively correlate with BMD in a number of investigations [29–32]. Despite the growing evidence that traditional anthropometric measurements are associated with BMD in several epidemiological studies, the obesity paradox still exists. The VAI, calculated using data from WC, TG, and HDL cholesterol, accurately reflects visceral fat distribution. Few studies have been conducted on VAI and BMD. DXA lumbar spine T-scores were positively correlated with VAI values, according to recent research [15]. Wung et al. also found a positive relationship between high VAI and high BMD, consistent with this study’s findings [33]. Although obesity benefits BMD, several studies have shown that obesity greatly increases an individual’s risk of developing conditions such as cancer and hypertension [34, 35]. This highlights the importance of maintaining VAI and BMD within an appropriate range.

Obesity and low BMD are linked, however, the underlying mechanism is still unclear. Several proposed mechanisms may contribute to this association. Firstly, excessive fat accumulation may increase the skeleton’s static mechanical compliance, leading to bone tissue alterations [36, 37]. Secondly, the growth of adipocytes in the bone marrow microenvironment may increase the production of chemicals that promote inflammation and regulate the immune system. These inflammatory chemicals may increase osteoclast formation and activation, reduce osteoblast differentiation, and stimulate osteoclasts [38]. Thirdly, overweight or obese individuals may synthesize and release higher amounts of insulin, estrogen, and other endocrine hormones, which help maintain BMD by preventing bone resorption and remodeling [39–42]. Fourthly, obesity may encourage bone mesenchymal stem cells (BMSCs) to differentiate into adipocytes, boosting the number of fat cells (adipocytes) and lowering the amount of bone-forming cells (osteoblasts) [43]. Finally, chronic inflammation in the fat tissue caused by obesity-related insulin resistance may be a factor in bone loss and decreased BMD, as adipose depots’ systemic production of inflammatory cytokines may contribute to this process [44, 45].

This study delved into the non-linear correlation compared to prior investigations, and the outcomes were just as surprising. A non-linear correlation was found between VAI and total femur BMD both before and after adjusting for confounding variables using a generalized additive model. This study is notable since it is the first to reveal a non-linear association between VAI and total femur BMD, including the discovery of threshold and saturation effects. This finding provides doctors with new tools to help people with obesity keep their VAI in a healthy range (about 3.3) and so preserve adequate BMD and lower their risk of obesity-related diseases and consequences. However, better explanation is needed as to the causes of the saturating effects of VAI on BMD. Why adult BMD does not rise after stunted growth may be due to the fact that bone development patterns and peak bone mass are set during early childhood [46, 47]. The presence of a separate bone-fat axis in vivo between adipose and bone tissue [48], coupled with numerous bioactive molecules that maintain bone homeostasis, is yet another factor in VAI saturation effects. According to the findings of researchers, bone and adipocytes have a common stem cell ancestor and compete with one another, with more fat leading to bone loss [49]. Experiments using animal models show that obesity, which is induced by high-fat diets, leads to a decline in BMD [50, 51].

### Study strengths and limitations

This research has numerous benefits, starting with the fact that the sample size is both representative and substantial. In fact, this study utilized the most extensive sample size related to this particular subject matter. Additionally, the research made careful adjustments for various confounding factors, ensuring that the findings are trustworthy and relevant to a diverse range of individuals. Furthermore, due to the high prices and radiation concerns of CT and the high prices and lengthy procedures of MRI, they are not appropriate for use in broad populations [52]. Therefore, this study efficiently investigates how visceral adiposity affects clinical outcomes by utilizing VAI. This computational model for calculating VAI considers anthropometric and physiological data to analyze adipose tissue distribution. However, it is also important to note that there are several limitations to the current research. The primary issue is that it is not possible to determine whether or not VAI caused the decline in total femur BMD. In addition, even after adjusting for a few probable confounders, the present study is still unable to totally exclude confounding brought on by certain unknown variables. Furthermore, this study did not examine additional practical assessment tools like FRAX in further detail due to technological considerations. Finally, this research’s study populations were wide-ranging and the findings may not be applicable to specific populations such as cancer patients, as they were not included in the present study.

## Conclusion

Ultimately, according to the results of the current research, there is a substantial positive association between VAI and BMD, with a saturation value determined for total femur BMD. The present study suggests that maintaining a moderate level of VAI (around 3.3) may let people over the age of 20 achieve the best possible VAI/BMD balance, promoting healthy bone growth. In times to come, VAI has the potential to assist individuals with obesity in upholding optimal BMD and reducing the risk of developing obesity-related diseases, thereby presenting a facile and cost-effective approach.

## Data Availability

The datasets analyzed during the current study are available on the NHANES official website, https://wwwn.cdc.gov/Nchs/Nhanes/.

## Abbreviations

VAI: Visceral adiposity index
BMD: Bone mineral density
NHANES: National Health and Nutrition Examination Survey
WC: Waist circumference
BMI: Body mass index
TG: Triglyceride
HDL: High-density lipoprotein
ALT: Alanine transaminase
ALP: Alkaline phosphatase
AST: Aspartate aminotransferase
SD: Standard deviation
GAM: Generalized additive model
WHO: World Health Organization
DXA: Dual-energy X-ray absorptiometry
BMSCs: Bone mesenchymal stem cells
